# Editorial: Host-pathogen interactions: the metabolic crossroads

**DOI:** 10.3389/fcimb.2023.1212051

**Published:** 2023-05-16

**Authors:** Crystal L. Richards, Sébastien Bontemps-Gallo, Travis J. Bourret

**Affiliations:** ^1^ Laboratory of Bacteriology, Division of Intramural Research, Rocky Mountain Laboratories, National Institute of Allergy and Infectious Diseases, National Institutes of Health, Hamilton, MT, United States; ^2^ Univ. Lille, CNRS, Inserm, CHU Lille, Institut Pasteur de Lille, U1019 - UMR 9017 - CIIL - Center for Infection and Immunity of Lille, Lille, France; ^3^ Department of Medical Microbiology and Immunology, Creighton University School of Medicine, Omaha, NE, United States

**Keywords:** metabolism, metabolites, microbiota, pathogens, infection

Host metabolism, and that of the microorganisms that colonize them, serves multiple vital roles in cellular biology and immune responses. The concentration and manipulation of metabolites can serve as potent signals regulating numerous processes during health and disease. Moreover, the presence of microorganisms - beneficial or not - can radically change the metabolism of the host, and the colonizing microorganisms, by directly influencing the metabolites present in the host. Innate and cellular immune responses to microorganisms are complex and interdependent processes that require multiple cell types to generate coordinated effects designed to control and sometimes, eliminate the colonizing agent. Any perturbations to these interactions, no matter how subtle, have the potential to “tip” the balance in favor of inflammation and development of disease.

In an important study by Que et al. the microbiome of the white-headed langur, a highly endangered species of monkey living in the Quangxi region of China, was compared to several other endemic species including François’ langur, silvered langur, loris, pygmy loris, ring-tailed lemur, macaques, gibbon, and baboon. Using metagenome analysis, it was shown that the microbiome of herbivorous monkeys had a metabolic signature consistent with a low-sugar diet, unlike omnivorous monkeys. Functional analysis revealed that energy metabolism, particularly, sugar metabolism-related pathways were less abundant in white-headed langurs and François’ langurs than in other primate species examined. These differences potentially point to metabolic energy conservation strategies as an adaptation to a primarily low-energy producing, leaf-eating diet. While the relationship between diet and microbiome is still poorly understood, this study represents another step towards understanding the role of environment and diet in the establishment and maintenance of the gut microbiome.

The impact of bacterial, viral or parasitic infection on the microbiome and its diversity has similarly remained poorly understood. However, it has been observed that the presence of pathogens can disrupt the fine balance of the microbiome that ensures cellular homeostasis. Exploring the consequences of *Plasmodium cynomolgi* infection in the rhesus macques model, Farinella et al. showed that bacterial community structures changed during *P. cynomolgi* infection, with the greatest impact observed during the first peak of the parasite infection, compared to subsequent relapses. Functional analysis of the gut microbiome revealed an increase in the genetic capacity to synthesize tryptophan, and an increase in kynurenine levels in the host. These changes appeared to be a consequence of an increase in the Helicobacteraceae family, an important opportunisitc pathogen of the stomach. The previously uncharacterized relationship between *Plasmodium cynomolgi* infection and Helicobacteraceae colonization merits further investigation and highlights how infection with one pathogen could promote the increased colonization of a completely separate and potentially pathogenic microorganism.

The role of metabolites in modulating disease severity has been extensively studied over the years and several important pathways have been identified to play a role in host inflammation and immune responses which subsequently determine continued pathogen colonization and persistence or clearance and recovery. In a review article on kynurenine synthesis, Oliveira et al. characterized the current state of research of this important metabolic pathway and focused on its role in two disparate disease states, leprosy and COVID-19. Studies showed that infection with *Mycobacterium leprae* or SARS-CoV-2 viral infection led to an increase in kynurenine, highlighting this metabolite as a potential broad marker for disease severity. Additionally, a role for kynurenine derivatives in neuroimmunomodulation has been revealed and Oliveira et al. suggested future strategies to characterize this metabolite in infectious diseases in general, as well as develop strategies to modulate kynurenine metabolism to through therapeutic strategies.

Another insightful review article by Groth et al. described the important role that endogenous oxidants play in modulating host cell function through lipid and protein modifications during tick-borne infections. Modification of cellular enzymes subsequently leads to changes in enzyme activity to alter metabolic activities and promote oxidative stress resistance. This article highlights the complexity of host-pathogen responses to host-produced oxidative stress conditions and how those interactions influence the evolution of disease pathophysiology.

Together these articles provide new insights into several poorly understood aspects of mammalian metabolism during health and disease. A deeper understanding of the role of diet and infection status in modulating the microbiome as well as the metabolic status of the host, will facilitate exploration of therapeutic strategies to treat infectious and non-infectious metabolic disorders.

## Author contributions

All authors listed have made a substantial, direct, and intellectual contribution to the work and approved it for publication.

